# Small-sized type A thymoma with pulmonary metastasis: a case report

**DOI:** 10.1186/s40792-022-01366-0

**Published:** 2022-01-20

**Authors:** Sachi Kawagishi, Tomohiro Maniwa, Hirokazu Watari, Ryuhei Sakata, Akiisa Omura, Ryo Tanaka, Toru Kimura, Keiichiro Honma, Jiro Okami

**Affiliations:** 1 Department of General Thoracic Surgery, Osaka International Cancer Institute, 1,3,4: 3-1-69, Otemae, Chuo-ku, Osaka, 541-8567 Japan; 2 Department of Pathology, Osaka International Cancer Institute, 1,3,4: 3-1-69, Otemae, Chuo-ku, Osaka, 541-8567 Japan

**Keywords:** Atypical type A thymoma, Thymus, Pulmonary metastasis, Anterior mediastinal tumor

## Abstract

**Background:**

Type A thymomas comprise a homogenous population of neoplastic epithelial cells that are characterized by a spindle/oval shape without nuclear atypia. They may be accompanied by few non-neoplastic lymphocytes. Most type A thymomas are detected in the earlier Masaoka stages. Compared to other thymoma subtypes, they rarely metastasize or recur. There have been some reports of patients with type A thymomas with pulmonary metastasis; however, these thymomas were 20 mm or more in size. Herein, we report the case of a patient who underwent surgical resection for a small-sized type A thymoma (12 mm) with pulmonary metastasis.

**Case presentation:**

A 62-year-old patient presented with an abnormal shadow in the left lung on plain chest radiography during a medical checkup. Chest computed tomography revealed a 12-mm tumor in the anterior mediastinum and a 13-mm nodule in the left lower lobe. ^18^F-fluorodeoxyglucose positron emission tomography/computed tomography revealed uptake in the anterior mediastinal tumor, but did not show a significant uptake in the pulmonary nodule. The patient underwent surgical resection on two separate occasions, and was diagnosed with an atypical type A thymoma and pulmonary metastasis. The TNM classification was p-T1aN0M1b stage IVb, and it was stage IVb according to the Masaoka staging system. No recurrence was observed during the follow-up.

**Conclusions:**

We report a case of the smallest type A thymoma with pulmonary metastasis. Pulmonary metastasis secondary to a type A thymoma should be considered even if the thymoma is small in size (< 20 mm).

## Background

Type A thymomas comprise a homogenous population of neoplastic epithelial cells that are characterized by a spindle/oval shape without nuclear atypia. They may be accompanied by a few non-neoplastic lymphocytes. Type A thymomas are associated with a favorable clinical course. Compared to other thymoma subtypes, they rarely metastasize or recur [1, 2]. However, some authors have reported cases of type A thymomas with pulmonary metastasis, wherein the thymomas were 20 mm or more in size [3–8]. We report the case of a patient who underwent surgical resection for a small-sized (12 mm) atypical type A thymoma and pulmonary metastasis.

## Case presentation

**Fig. 1:**
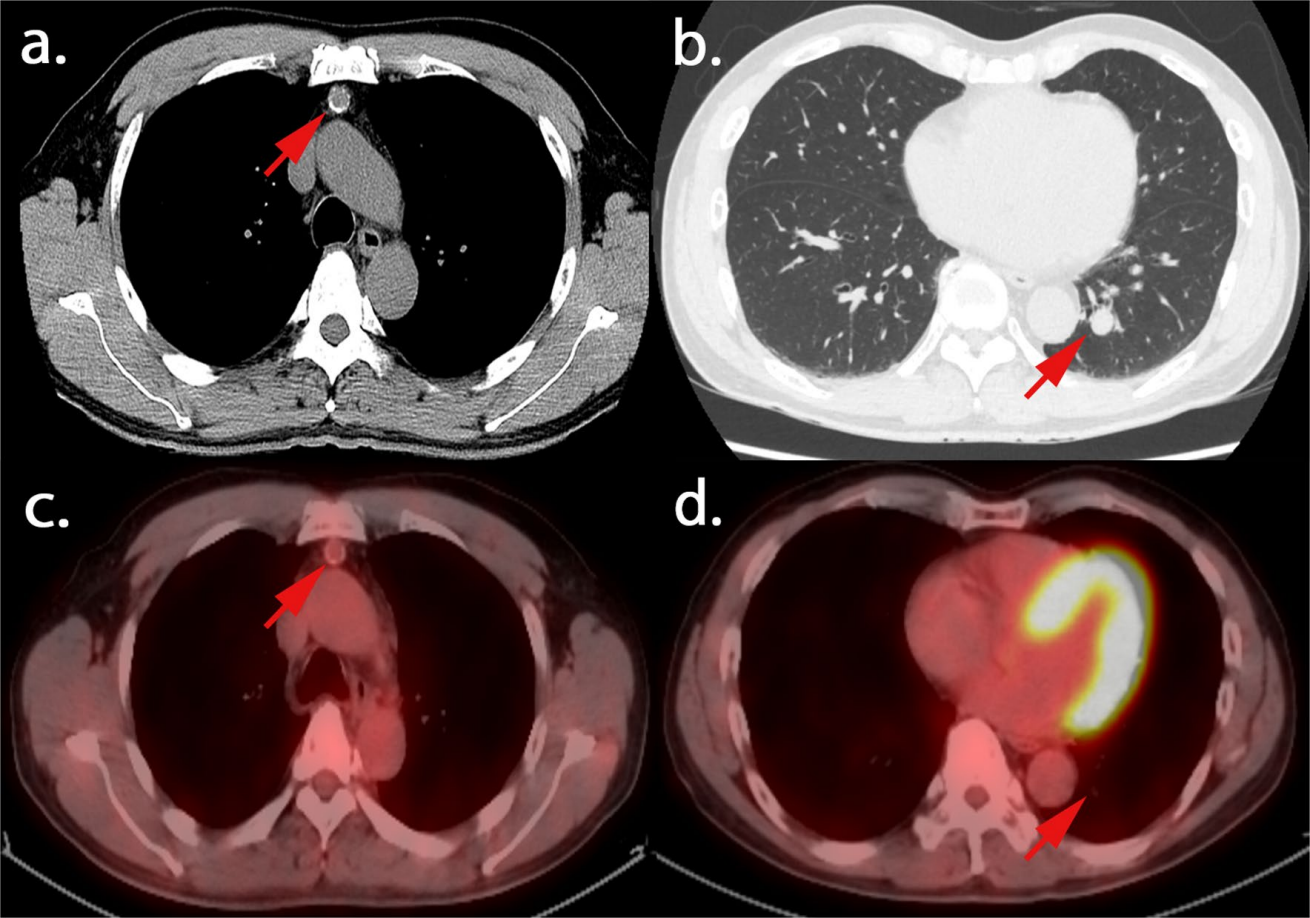
**a**, **b** Chest computed tomography findings. A tumor with calcification is located in the anterior mediastinum (**a**) and a well-defined hilar nodule is located in S^10^ of the left lung (**b**). **c**, **d** ^18^F-fluorodexyglycose positron emission tomography/computed tomography findings. Uptake is noted in the anterior mediastinal tumor (SUV_max_ = 1.5) (**c**), but no significant uptake is noted in the pulmonary nodule (SUV_max_ = 1.1) (**d**)

**Fig. 2:**
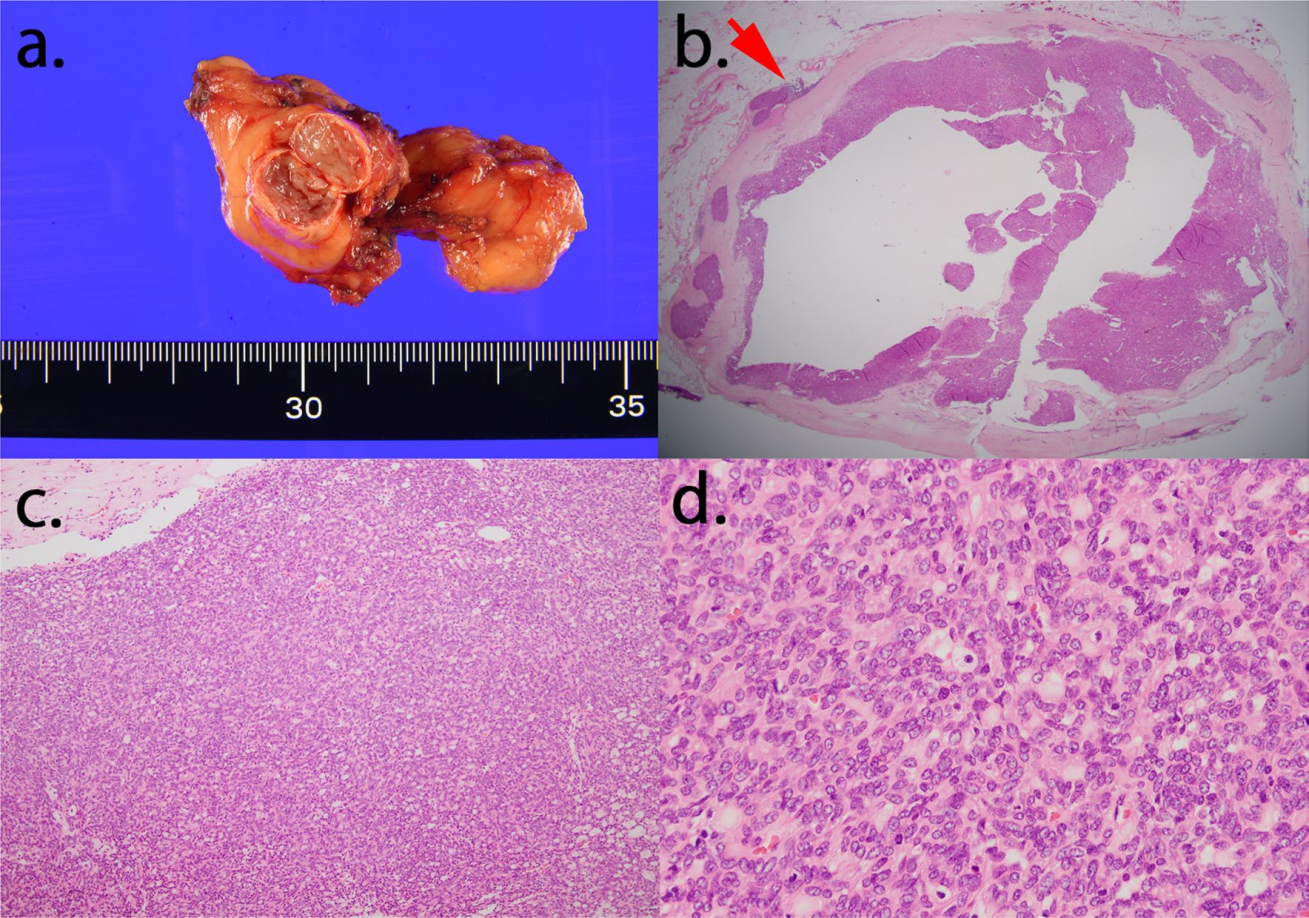
**a** Macroscopic image of the excised thymic tumor. **b**–**d** Hematoxylin and eosin staining of the anterior mediastinal tumor. Microscopic invasion into the surrounding adipose fatty tissue beyond the capsular portion is noted only in one part of the tumor (**b**, arrow). Tumor cells with relatively poor atypia and round nuclei are seen to grow in a fascicular pattern. The tumor shows findings of hypercellularity (**c**, **d**)

A 62-year-old man presented with an abnormal shadow in the left lung on plain chest radiography during a medical checkup. Chest computed tomography (CT) revealed a nodule in the left lower lobe and a tumor with calcification in the anterior mediastinum. The well-defined hilar nodule of the left lung measured 13 mm and was located in S^10^. The anterior mediastinal tumor measured 12 mm in size, and there was no evidence of infiltration into the adjacent tissue (Fig. 1a, b). While ^18^F-fluorodeoxyglucose positron emission tomography/CT (FDG-PET/CT) revealed uptake in the anterior mediastinal tumor (SUV_max_ = 1.5), it did not reveal significant uptake in the left lower-lobe nodule (SUV_max_ = 1.1) (Fig. 1c, d). The patient was seronegative for anti-acetylcholine receptor-binding antibodies and tumor markers. The bronchoscopy was negative for pulmonary nodule, and FDG-PET/CT did not reveal significant uptake. From these findings, the pulmonary nodule was suggested to be a benign tumor. Surgical resection for diagnostic treatment was planned for the anterior mediastinal tumor, and pulmonary resection was planned for later. Contrast-enhanced chest CT revealed that the anterior mediastinal tumor was adjacent to the left brachiocephalic artery, and the border between them was unclear; therefore, the possibility of the adhesions between the tumor and artery was considered. Thymectomy was performed via a median sternotomy in consideration of safety. The operation time was 134 min and the bleeding volume was 75 mL. The tumor measured 16 mm × 13 mm × 9 mm. The postoperative course was uneventful, and the patient was discharged 7 days after the operation. The pathological diagnosis was of an atypical type A thymoma. Pathological examination revealed that the tumor cells exhibited poor atypia, had round nuclei, and grew in a fascicular pattern. The tumor had a hemangiopericytoma-like vascular pattern. It also had hypercellularity, but no increased mitotic counts and focal necrosis were observed. The tumor was almost encapsulated by the fibrous cap. Microscopic invasion into the surrounding adipose fatty tissue beyond the capsular portion was noted in one part of the tumor (Fig. 2).

**Fig. 3:**
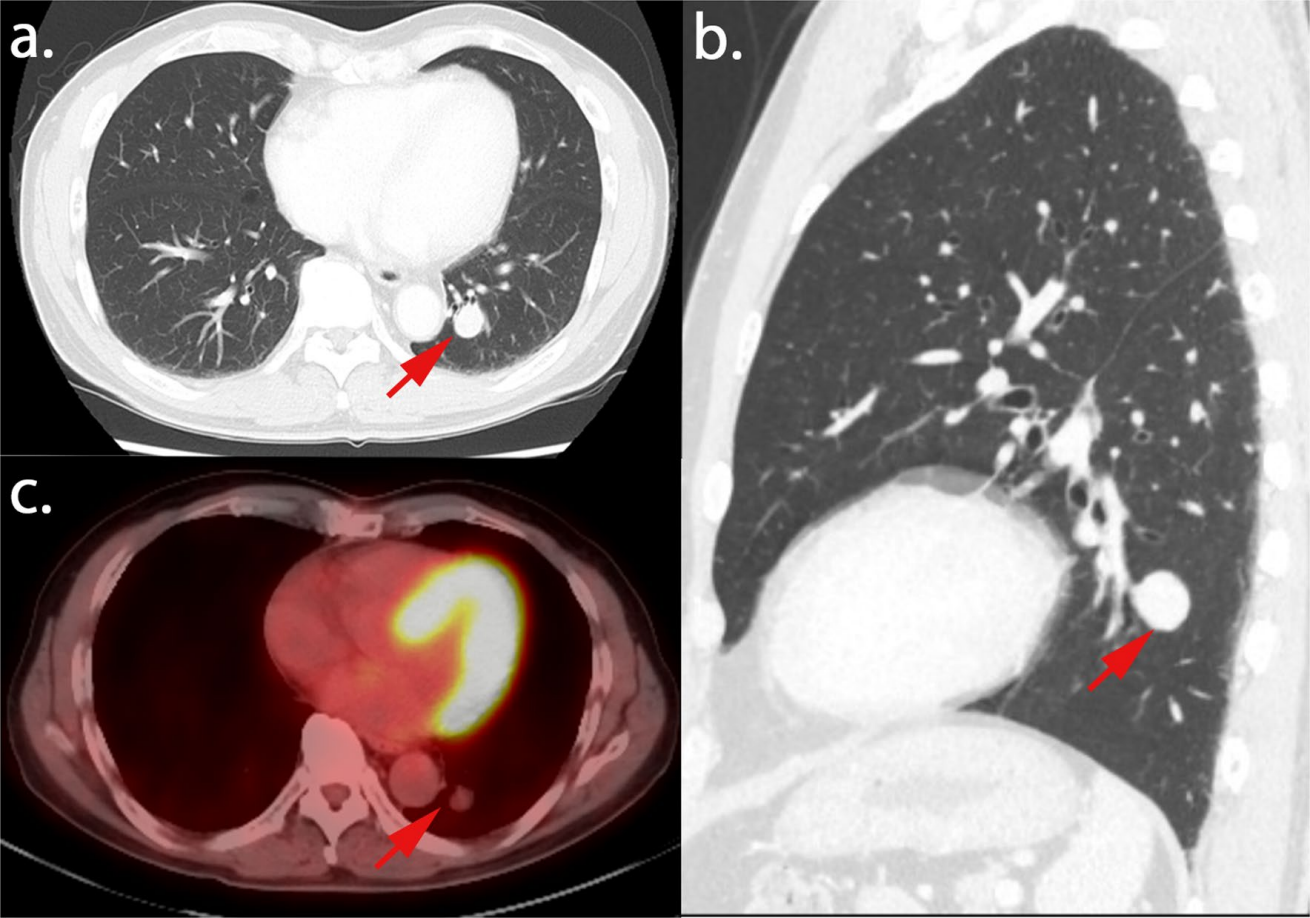
**a**, **b** Chest computed tomography findings. The nodule of the left lower lobe increased in size, and it was near the segmental bronchus. **c** ^18^F-fluorodexyglycose positron emission tomography/computed tomography reveal a little uptake (SUV_max_ = 1.3)

**Fig. 4:**
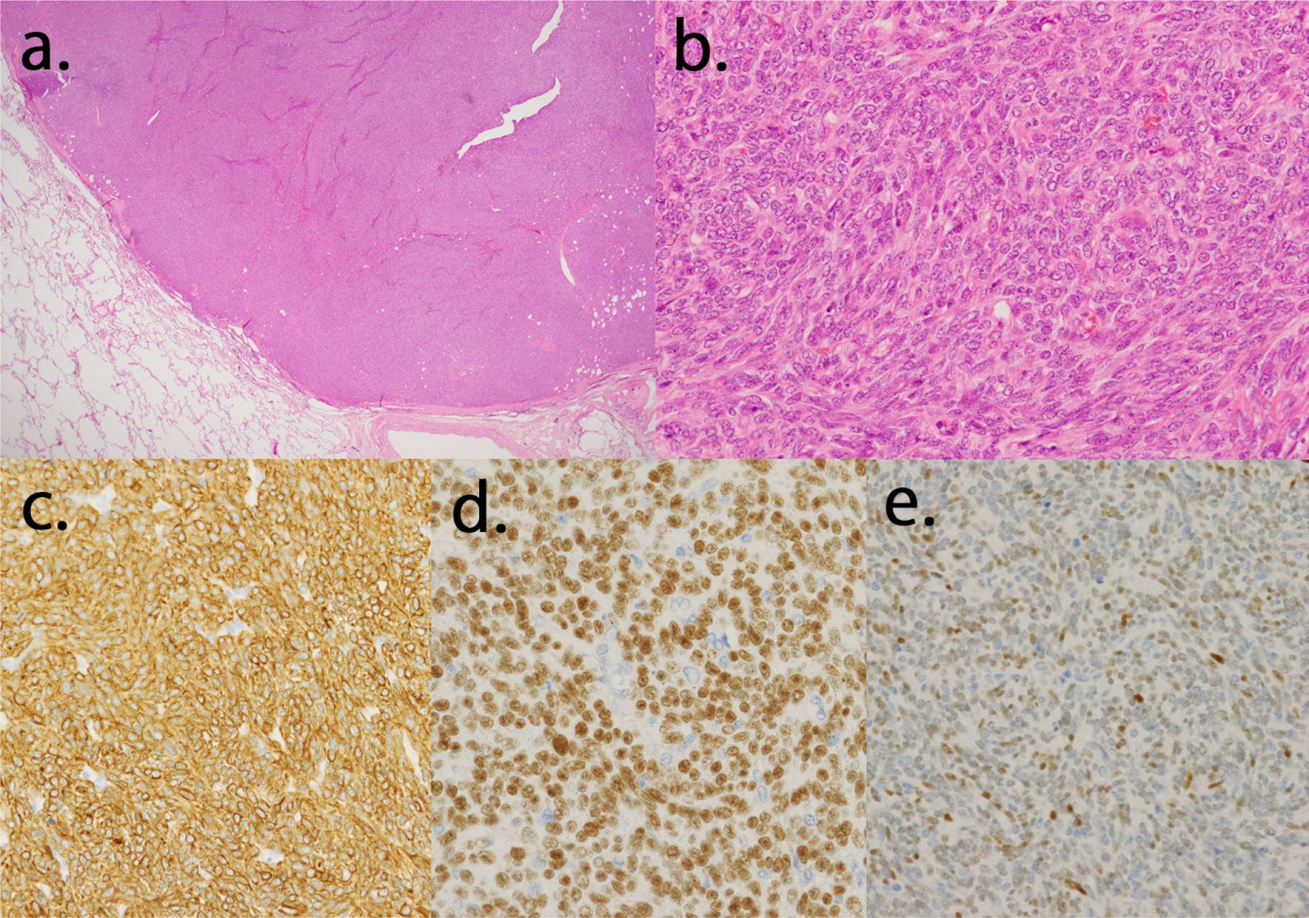
**a**, **b** Hematoxylin and eosin staining of the pulmonary nodule. Pathological images are similar to those of an anterior mediastinal tumor. **c**–**e** An immunohistochemical analysis of the pulmonary disease. The tumor is noted to be positive for the anti-pan cytokeratin antibody (**c**), p40 (**d**), and paired box protein 8 (**e**)

A pulmonary nodule resection was planned after the surgery; however, the patient refused to undergo it because of his poor physical condition. Fourteen months after the surgery, follow-up chest CT revealed that the left lower-lobe nodule had enlarged to 18 mm (Fig. 3a, b). FDG-PET/CT revealed a slight uptake (SUV_max_ = 1.3) in the nodule. The nodule itself was considered a low-grade malignant tumor (Fig. 3c). Surgical resection for diagnostic treatment was planned to rule out malignancy. Sixteen months after the initial surgery for the thymoma, the patient underwent left lower lobe lobectomy and lymph node dissection. The nodule of the left lower lobe was near the segmental bronchus (Fig. 3b). We considered that we could not take a sufficient resection margin of the tumor if we performed sublobar resection. The operation time was 190 min, and the bleeding volume was 20 mL. The tumor measured 13 mm × 12 mm × 8 mm. The postoperative course was uneventful, and the patient was discharged 8 days after the operation. Postoperative pathological findings revealed densely scattered spindle cells. Immunohistochemical analysis revealed that the tumor cells were positive for the anti-pan cytokeratin antibody, p40, and paired box protein 8. The tumor exhibited hypercellularity. However, the mitotic count was not increased, and focal necrosis was not observed (Fig. 4). The tumor was diagnosed as a metastasis of the atypical type A thymoma. The TNM classification was p-T1aN0M1b stage IVb, and it was stage IVb according to the Masaoka staging system. The tumor was completely resected, and the patient did not receive adjuvant therapy. The patient experienced no recurrence for 21 months after the surgery for thymoma and for 5 months after the pulmonary resection.

## Discussion

Thymomas originate from the epithelial cells of the thymus. These tumors are unique due to their ability to partially promote T-lymphocyte maturation. Moreover, no anaplastic cells were noted in the present case. In case of type A thymomas, the occurrence of metastases and tumor-related death are generally related to high-stage disease (Masaoka stages III and IV) and incomplete tumor resection [1, 9]. In the previously reported cases of type A thymomas, detection was during the earlier Masaoka stages (Masaoka stages I and II) and the tumors rarely metastasized [1]. The atypical type A thymoma variant was added to the World Health Organization classification as a new subtype in 2015. It may be associated with postoperative recurrence or distant metastases [10].

Previous reports have described pulmonary metastases of type A thymomas along with the thymoma sizes (Table 1) [3–8]. In these cases, there were almost no differences between the typical and atypical type A thymomas. Despite thymoma invasion, these patients had pulmonary metastasis. In previous cases of type A thymomas with pulmonary metastasis, the primary tumors were larger than 20 mm. However, in the present case, the primary tumor measured 12 mm in size, and the metastatic tumor was larger than the thymoma.

**Table 1:** Previous reports that describe a type A thymoma with pulmonary metastasis and thymoma size

Case no.	Author	Year	Age (years)	Sex	Pathology of the thymoma	Size of the thymoma (mm)	Size of the pulmonary metastasis (mm)	Invasion of the thymoma
1	Hirono et al. [3]	2014	74	M	–	30	20	None
2	Hashimoto et al. [4]	2017	35	F	Atypical	60	–	None
3	Burger et al. [5]	2017	72	M	Typical	70	–	Mediastinal fat
4			64	M	Typical	28	20	–
5			66	M	Atypical	70	–	–
6	Mengoli et al. [6]	2017	55	M	–	44	8	Fibrous capsule
7	Kawakita et al. [7]	2018	84	M	Atypical	44	2–5	Vascular
8	Tatematsu et al. [8]	2021	79	F	Typical	21	3	–
9	Our case	2021	62	M	Atypical	12	13	Fibrous capsule

*M* male, *F* female

This report presents a case of the smallest type A thymoma with pulmonary metastasis reported yet. Due to the smaller size of the thymoma, the pulmonary nodule was not identified as a metastatic lesion. In cases wherein the type A thymoma measures less than 20 mm, pulmonary metastasis should still be considered. Complete surgical resection is the most important prognostic factor in thymomas with local infiltration or metastasis [11]. Therefore, aggressive resection is warranted in cases wherein the thymoma and pulmonary metastasis can be resected completely. Considering the possibility of recurrence, a long-term follow-up is necessary.

## Conclusions

We report the case of a patient who underwent surgical resection for an atypical type A thymoma and solitary pulmonary metastasis. In cases of a type A thymoma with a pulmonary nodule, pulmonary metastasis secondary to the thymoma should be considered even when the type A thymoma measures less than 20 mm, as in our case.

## Data Availability

The patient data of this case report will not be shared to ensure patient confidentiality.

## Abbreviations

CT: Computed tomography
FDG-PET: ^18^F-fluorodeoxyglucose positron emission tomography
